# Enamel Maturation as a Systems Physiology: Ion Transport and Pi Flux

**DOI:** 10.3390/cells14221821

**Published:** 2025-11-20

**Authors:** Mehrnaz Zarinfar, Marziyeh Aghazadeh, Rucha Arun Bapat, Yanbin Ji, Michael L. Paine

**Affiliations:** Center for Craniofacial Molecular Biology, Department of Biomedical Sciences, Herman Ostrow School of Dentistry of USC, University of Southern California, Los Angeles, CA 90089, USA

**Keywords:** phosphate transport, amelogenesis, amelogenesis imperfecta, SLC34A2 (NaPi-IIb), SLC20A1 (PiT1), SLC20A2 (PiT2), XPR1

## Abstract

Dental enamel, the final product of amelogenesis, is a highly mineralized bioceramic that becomes acellular and non-regenerating after tooth eruption. This paper reviews literature that explores inorganic phosphate (Pi) transport during the process of enamel formation or amelogenesis. Evidence from transcriptomics, immunolocalization, and physiology implicates ameloblast-specific sodium-dependent Pi uptake by type III sodium–phosphate cotransporters SLC20A1 (PiT1) and SLC20A2 (PiT2), and by type IIb sodium–phosphate cotransporter SLC34A2 (NaPi-IIb) with stage-specific basal (proximal) or apical (distal) enrichment, and pH-dependent expression. Controlled Pi efflux to the enamel space has been partly attributed to xenotropic and polytropic retrovirus receptor (XPR1) mediated Pi export during maturation-stage amelogenesis. These amelogenesis-specific Pi fluxes operate within a polarized cellular framework in which Ca^2+^ delivery and extrusion, together with bicarbonate-based buffering regulated by cystic fibrosis transmembrane conductance regulator (CFTR), Solute carrier family 26 (SLC26) exchangers, anion exchanger 2 (AE2), and electrogenic sodium bicarbonate cotransporter 1 (NBCe1), at-least partially contribute to cellular Pi activity, and neutralize protons generated as the extracellular hydroxyapatite-based enamel matures. Disruption of phosphate handling reduces crystal growth and final mineral content of enamel, and produces hypomineralized or hypomature enamel with opacities, post-eruptive breakdown, and greater caries susceptibility. This review integrates multi-modal findings to appraise established features of ameloblast Pi handling, define constraints imposed by pH control and Ca^2+^ transport, and identify gaps in ion transporter topology and trafficking dynamics.

## 1. Introduction

Dental enamel, the hardest and most mineralized tissue in the human body, is formed through a highly regulated process called amelogenesis [1]. Once enamel formation is complete and the tooth erupts, the tissue becomes acellular and incapable of regeneration, meaning that any developmental disturbance is permanently preserved in the mature enamel layer [2]. Amelogenesis proceeds in distinct stages of secretory, transition, and maturation, during which ameloblasts orchestrate extracellular matrix deposition, protein processing, and massive influx and efflux of ions necessary for hydroxyapatite crystal growth [3,4,5,6].

The transport and regulation of calcium, chloride, sodium, potassium, and magnesium in ameloblasts are well-documented, with comprehensive reviews detailing channel and transporter repertoires across secretory and maturation stages (e.g., Ca^2+^/Store-Operated Calcium Entry (SOCE), potassium-dependent sodium-calcium exchanger (NCKX)/sodium-calcium exchanger (NCX)/Plasma Membrane Calcium ATPase (PMCA); CFTR/AE2/NBCe1; Na+/K+-adenosine triphosphatase (NKA)/sodium/hydrogen exchanger 1 (NHE1)/K^+^ channels; cyclin and CBS domain divalent metal cation transport mediator 4 (CNNM4)/Transient receptor potential cation channel, subfamily M, member 7 (TRPM7)) [1,7]. By contrast, inorganic phosphate (Pi), a fundamental building block of hydroxyapatite, has historically received less focused attention in enamel biology, despite classic tracer studies showing Pi incorporation during enamel development [8,9]. Only recently has the field begun to define how ameloblasts regulate Pi uptake, distribution, and efflux. Sodium-dependent phosphate transporters SLC20A1/SLC20A2 (PiT1/PiT2) and SLC34A2 (NaPi-IIb), together with the exporter XPR1, show stage-resolved expression in the enamel organ. Recent structural work also establishes XPR1 as the dedicated mammalian phosphate exporter, refining models of Pi handling at the enamel surface [9,10,11]. These findings point to a more complex and tightly coordinated phosphate transport network than previously recognized [1,7,12]. Understanding these mechanisms is crucial, as disruptions in phosphate handling may directly interfere with mineralization, leading to structural defects in enamel.

## 2. Overview of Amelogenesis

Amelogenesis is the complex, highly regulated process by which dental enamel, the most mineralized and hardest tissue in the vertebrate body, is formed by specialized epithelial cells called ameloblasts [1,13,14,15]. This process is confined to the enamel organ and proceeds in concert with tooth morphogenesis, ultimately producing a non-collagenous, acellular, and avascular mineral layer that, once matured, cannot regenerate [2,14,16].

Tooth development begins with the dental lamina, a thickened band of oral epithelium that invaginates into neural crest-derived mesenchyme to form the dental placode [17,18]. Through the bud, cap, and bell stages, reciprocal epithelial–mesenchymal signaling orchestrates crown morphogenesis [19,20,21]. During the bell stage, the enamel organ differentiates into the inner enamel epithelium (precursor to ameloblasts), outer enamel epithelium, stellate reticulum, and stratum intermedium [13,22]. Inductive cues from the underlying dental mesenchyme not only trigger ameloblast differentiation but also guide the morphogenesis of the dentin-enamel junction (DEJ), which sets the blueprint for enamel thickness and shape [23].

Amelogenesis is traditionally divided into three functional stages, secretory, transition, and maturation, preceded by a pre-secretory stage [1,24,25]. Each stage is marked by distinctive morphological and molecular features that reflect changing ameloblast functions: matrix production, ion transport, and matrix removal. Some authors further subdivide these into early/late phases, but the central functional transition remains between secretion and maturation [26,27,28,29].

Because enamel formation proceeds without subsequent remodeling, any disruption, whether genetic, environmental, or systemic, can lead to permanent defects such as amelogenesis imperfecta, fluorosis, or hypomineralization [1,30]. Although ameloblasts may partially compensate for transient disturbances, fully matured enamel is irreversible and lacks intrinsic repair capacity [4,30]. A stage-specific understanding of ameloblast biology is essential not only for developmental biology but also for designing regenerative and biomimetic repair strategies.

### 2.1. Pre-Secretory Stage

The pre-secretory stage marks the earliest commitment of inner enamel epithelial cells to the ameloblast lineage, occurring after odontoblast differentiation and the initial deposition of predentin. This dentin matrix serves as a critical trigger for ameloblast polarization and cytodifferentiation [31,32,33,34,35,36,37]. During this phase, pre-ameloblasts elongate, reverse nuclear polarity (shifting nuclei away from the basement membrane), and reorganize their cytoskeleton. These morphological changes transform undifferentiated epithelial cells into highly polarized units capable of specialized secretion in the next stage [3,37,38,39]. This transition is driven by tightly coordinated epithelial–mesenchymal interactions. Reciprocal epithelial–mesenchymal signals, including bone morphogenetic proteins (BMPs), fibroblast growth factors (FGFs), transforming growth factor-beta (TGF-β), sonic hedgehog (Shh), and Wingless-related integration site (Wnt) ligands, activate transcriptional programs in pre-ameloblasts that initiate expression of enamel matrix genes such as *Ameloblastin (AMBN)*, *Enamelin (ENAM)*, and *Amelogenin X-linked (AMELX)*. These signals initiate low-level expression of AMBN, ENAM, and AMELX (with incipient Matrix metallopeptidase 20 (MMP20)) at the pre-secretory stage that is detectable at mRNA and protein levels but below the robust expression characteristic of the ensuing secretory stage [1,40,41,42,43,44,45,46].

AMBN protein is particularly prominent in the pre-secretory and early secretory stages, functioning in cell–matrix adhesion to maintain contact between newly polarized ameloblasts and the forming dentin matrix [13,47,48,49]. Supporting layers of the enamel organ start to assume their specialized roles. The stratum intermedium expresses alkaline phosphatase (ALPL) and other factors thought to facilitate phosphate metabolism and early ion handling, while the stellate reticulum maintains the spatial organization of the enamel organ via an extracellular matrix rich in glycosaminoglycans [1,50,51,52].

**Figure 1:**
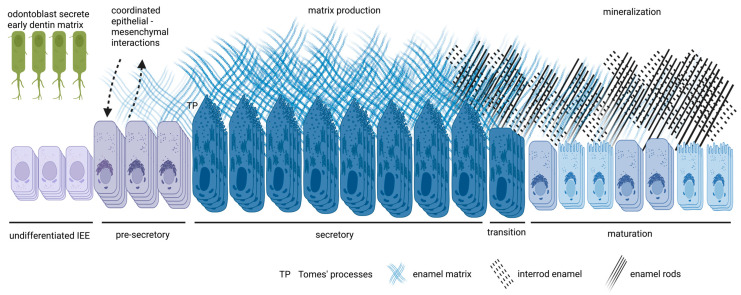
Schematic showing an overview of amelogenesis from the inner enamel epithelium (IEE) to the maturation stage. From left to right, signals from odontoblasts induce the differentiation of IEE cells into pre-ameloblasts. This developmental phase, also referred to as the pre-secretory stage, is characterized by tightly regulated epithelial–mesenchymal interactions that culminate in the formation of secretory ameloblasts. Secretory ameloblasts develop a highly specialized apical extension, the Tomes’ process, and initiate secretion of the enamel extracellular matrix (depicted in blue, apical to the Tomes’ processes). The subsequent transition stage represents a brief but critical shift from matrix secretion to mineralization. Enamel matrix proteins are progressively removed and replaced by hydroxyapatite that assemble into the distinctive rod-interrod architecture (represented by black solid and dashed lines). A hallmark of the maturation stage is the alternation between two ameloblast morphologies, ruffle-ended and smooth-ended, visualized here as alternating groups of cells with ruffled versus smooth apical surfaces.

Although mineralization has not yet begun, pre-secretory ameloblasts assemble the biosynthetic machinery for secretion through expanding their rough endoplasmic reticulum and Golgi apparatus and generating secretory vesicles. By the end of this stage, the architectural and molecular groundwork is in place for large-scale matrix production (Figure 1) [25,34,37,53].

### 2.2. Secretory Stage

The secretory stage is the first major functional phase of enamel formation, during which ameloblasts establish the full thickness of the enamel layer and the organizational framework for its rod-interrod architecture [36,54,55,56]. Ameloblasts in mammals become tall (~70 μm), columnar, and highly polarized, extending apical projections known as Tomes’ processes. These processes secrete enamel matrix in spatially distinct domains, producing the tightly interlocking prism (rod) and interrod pattern that confers mechanical strength and resistance to crack propagation [1,5,56].

The enamel matrix at this stage is composed primarily of three proteins. Amelogenin (AMELX) (~90% of the organic matrix), which self-assembles into ~20 nm nanospheres that align hydroxyapatite ribbons and prevent lateral fusion, producing the high aspect ratio characteristic of enamel crystallites. AMBN, which sustains cell–matrix adhesion and ameloblast differentiation, ensuring precise cell positioning during secretion, and ENAM, which is essential for crystal nucleation and elongation [41,47,48,57,58,59,60]. These proteins create a scaffold that controls crystal orientation, growth, and spacing. Controlled proteolysis begins immediately with MMP20, which degrades matrix proteins in a regulated fashion to allow crystallite elongation while maintaining structural stability [61,62,63,64].

Initially, enamel is a partially mineralized, hydrated tissue with roughly equal proportions of mineral, protein, and water. Hydroxyapatite crystallites elongate along their c-axis from the dentin-enamel junction (DEJ) toward ameloblasts under near-neutral pH conditions. Evidence suggests nucleation may occur on mineralized dentin collagen fibrils at the DEJ, with enamel crystals extending outward into the protein scaffold [1,63,65,66]. Ameloblasts maintain the ionic microenvironment of the enamel space through tight apical junctions and more permeable basal junctions that allow nutrient and ion exchange with the vascularized supporting cell layers (stratum intermedium, stellate reticulum, and outer enamel epithelium). These layers are metabolically active, facilitating delivery of Ca^2+^, PO_4_^3−^, and other ions [1,6,67,68].

Enamel secretion proceeds in circadian and longer-period rhythms, recorded as cross-striations (~4 μm/day in humans) and striae of Retzius (~7–11 days). These incremental lines provide permanent records of secretion rate and developmental stress [69,70,71,72,73,74,75]. In continuously growing teeth such as rodent incisors, stem cells at the cervical loop maintain lifelong secretory activity, whereas in humans, ameloblasts terminally differentiate, restricting enamel production to a defined developmental window [1,76,77,78,79].

Transcriptomic studies (e.g., Postnatal day 5 (PN5) mouse molars) show high expression of *Amelx, Ambn, Enam, Mmp20,* and regulators of vesicle trafficking, cytoskeletal organization, and cell adhesion. Ion transporters are expressed at low levels in this stage and become more functionally significant during the transition and maturation stages [29,43,80,81,82].

In summary, the secretory stage constructs the proteinaceous scaffold that determines enamel thickness, prism organization, and initial mineral framework, setting the stage for the extensive mineralization and matrix clearance that follow (Figure 1).

### 2.3. Transition Stage

The transition stage is a brief yet pivotal period in amelogenesis, marking the shift from matrix production to mineralization [1,4,6,36,83]. In the rat lower incisor, it spans approximately 170 μm, equivalent to about 30 to 40 ameloblast widths, but in this short distance, the enamel organ undergoes extensive structural and molecular remodeling [1,4,6,84].

Ameloblasts that were tall and columnar during the secretory stage shorten from about 70 to 40 μm and retract their Tomes’ processes. At the same time, the enamel organ collapses from its multilayered form into a condensed papillary layer that replaces the previous organization of ameloblasts, stratum intermedium, stellate reticulum, and outer enamel epithelium. This papillary layer is highly vascularized and is strategically positioned to supply calcium, phosphate, and bicarbonate to the enamel surface during the upcoming maturation phase [1,3,84,85,86,87,88,89,90].

At the transcriptomic level, there is a pronounced switch in gene expression. Enamel matrix protein genes such as *Amelx*, *Ambn*, and *Enam* are markedly downregulated, while genes involved in ion transport, pH regulation, and proteolysis are strongly upregulated. Kallikrein-related peptidase 4 (Klk4) becomes increasingly active, complementing residual MMP20 activity to initiate the degradation of the remaining organic matrix. Several key transporters, including Solute carrier family 24 member 4 (Slc24a4), Ae2, Cftr, Nbce1, and carbonic anhydrase II (CA II), are upregulated at this point to prepare ameloblasts for the ion-handling demands of maturation [1,80,81,87,91,92,93,94].

A distinctive feature of transition is programmed cell death in a substantial fraction of ameloblasts (≈25%), a change documented in rodent incisors and linked to stage progression [95]. Mechanistically, apoptosis in this window has been associated with calcium overload and Endoplasmic reticulum (ER) stress (including fluoride-triggered ER stress in vivo and in ameloblast models) and with Transforming Growth Factor Beta 1 (TGF-β1)/SMAD family member 2 and 3 (Smad2/3) signaling, which is upregulated during late development and influences ameloblast survival and differentiation [96,97,98,99]. It may serve as a quality control step, ensuring that only fully functional ameloblasts proceed to the maturation stage. Odontogenesis-associated phosphoprotein (ODAPH), which peaks in expression during this phase, may help stabilize ameloblast adhesion during the structural reorganization of the enamel organ [97,98,100,101,102].

Overall, the transition stage prepares the enamel organ for the high metabolic demands of maturation (Figure 1). It achieves this by reorganizing epithelial architecture, priming ion transport systems, initiating matrix clearance, and selectively retaining the cells best equipped to complete enamel mineralization.

### 2.4. Maturation Stage

The maturation stage is the final and most mineralization-intensive phase of amelogenesis. During this period, enamel mineral content increases from around 30 percent to more than 95 percent by weight, while water and organic material are reduced to less than 2 percent. This transformation produces the dense, highly crystalline hydroxyapatite structure that gives enamel its exceptional hardness and resistance to wear [1,6,103,104,105,106].

Ameloblasts at this stage shorten to roughly 40 μm in height and cycle between ruffle-ended and smooth-ended morphologies (Figure 1). Ruffle-ended cells have a highly folded apical border, tight junctions, and a mitochondria-rich cytoplasm, creating a sealed environment optimized for active ion transport, pH regulation, and the endocytosis of protein fragments. Smooth-ended cells, by contrast, have a less complex apical surface and looser junctions, allowing greater fluid exchange that aids in the removal of degraded proteins and the dissipation of acidic byproducts. The cyclical modulation between these morphologies is thought to be driven by local pH fluctuations in the enamel space, with acidification promoting the ruffle-ended form and alkalinization favoring the smooth-ended form [1,4,6,24,36,85,107].

KLK4 activity reaches its peak during maturation, completing the degradation of amelogenin and other residual matrix proteins. This clearance is essential to create the space needed for crystals to grow laterally, increasing in width and thickness until they form densely packed prisms. The papillary layer, established during the transition stage, continues to supply calcium, phosphate, and bicarbonate to ameloblasts, while the stratum intermedium and stellate reticulum maintain phosphate regulation and structural support [6,55,88,108,109,110,111,112].

The functional details of ion transport and acid-base regulation at these stages are discussed extensively in the next section (Ion Transport During Enamel Maturation). Together, these processes ensure that the enamel reaches its full mineral density, crystal organization, and mechanical performance, completing a process that began with the first deposition of matrix at the dentinoenamel junction.

### 2.5. Molecular Regulation and Signaling

Amelogenesis is controlled by a complex network of signaling pathways that operate in a stage-specific manner to direct ameloblast differentiation, cytoskeletal organization, enamel matrix secretion, ion transport, and apoptosis. These pathways mediate reciprocal communication between the dental epithelium and the underlying mesenchyme, ensuring that gene expression and cellular behavior are precisely coordinated throughout enamel development. Disruptions in these molecular signals can produce developmental enamel pathologies, including various forms of amelogenesis imperfecta (AI) [1,25,44,45,46,113].

The Wnt/Catenin beta-1 (β-catenin) pathway plays a pivotal role during early enamel development, influencing ameloblast polarization, cytoskeletal organization, and transcriptional regulation. Activation of Wnt signaling stabilizes cytoplasmic β-catenin, enabling its translocation to the nucleus, where it modulates the transcription of epithelial differentiation genes. β-catenin also functions as part of the Epithelial cadherin (E-cadherin)/β-catenin complex at adherens junctions, maintaining epithelial integrity while transmitting intracellular signals that govern ameloblast fate, elongation, and spatial organization [1,114,115,116,117,118].

TGF-β1 signaling becomes particularly important during the transition and maturation stages. Through the canonical Smad2/3 pathway, it modulates the expression of key enamel matrix genes, including *AMELX*, *AMBN*, *ENAM*, *MMP20*, and *KLK4*. In addition to regulating matrix gene transcription, TGF-β1 appears to promote ameloblast apoptosis during the transition stage, potentially serving as a checkpoint to eliminate cells that are damaged or improperly polarized [98,119,120,121,122,123]. This dual role, supporting both matrix production and programmed cell death, underscores the highly stage-specific function of TGF-β1 in amelogenesis.

Members of the bone morphogenetic protein family, particularly Bone morphogenetic protein 2 (BMP2) and Bone morphogenetic protein 4 (BMP4), are similarly essential for initiating ameloblast lineage commitment and sustaining enamel matrix gene expression during the secretory stage. BMP signaling interacts with TGF-β pathways through shared Smad proteins and cooperates in regulating epithelial–mesenchymal signaling networks. These interactions also influence fibroblast growth factor receptor 1 (FGFR1) expression, linking morphogen signaling to cell proliferation and differentiation [32,79,124,125,126].

Fibroblast growth factor signaling, especially through FGFR1, contributes to ameloblast proliferation, spatial organization, and cytoskeletal stability. Downstream activation of mitogen-activated protein kinase (MAPK) cascades, particularly p38 MAPK, appears to regulate polarity proteins and vesicular trafficking mechanisms necessary for the directional secretion of matrix protein [36,125,127,128,129].

Sonic hedgehog signaling is highly active during the secretory stage, regulating ameloblast proliferation, alignment, and the morphogenesis of the enamel organ. It also influences the expression of genes involved in vesicle trafficking and polarity establishment, and it interacts with Wnt and BMP pathways to fine-tune epithelial morphodynamics, particularly during cusp formation [36,46,130,131,132,133].

In addition to these canonical signaling systems, several matrix-associated proteins and transcription factors act as regulatory intermediates. Ameloblastin not only serves as a matrix protein but also acts as a signaling molecule that influences ameloblast adhesion, polarity, and actin cytoskeleton organization. The transcription factor Runt-related transcription factor 2 (RUNX2), better known for its role in osteoblast and odontoblast differentiation, also participates in enamel formation by regulating MMP20 expression and matrix remodeling. Odontogenic ameloblast-associated protein (ODAM), expressed during the transition and maturation stages, supports ameloblast attachment to the enamel surface and may modulate integrin-mediated signaling cascades [48,134,135,136,137].

Together, these pathways form an integrated regulatory network that coordinates gene expression, cell morphology, and functional activity at each stage of amelogenesis. A detailed understanding of these mechanisms not only provides insight into developmental biology but also offers potential targets for tissue engineering and the design of biomimetic strategies for enamel regeneration.

### 2.6. Clinical Implications and Enamel Defects

Because enamel formation is confined to a brief developmental window and depends entirely on transient ameloblast activity, any disturbance during secretion or maturation becomes permanently recorded in the tissue. Once erupted, enamel is acellular and lacks remodeling capacity, so these defects cannot be repaired [5]. The nature of the defect depends on the timing, duration, and type of insult, as well as the developmental stage affected [138]. Disturbances in matrix secretion during the secretory stage produce hypoplasia, characterized by reduced enamel volume, pits, or grooves. Disruptions during the maturation stage impair crystal growth and protein clearance, producing hypomineralized or hypomature enamel that is often chalky, porous, and mechanically weak [6,139,140,141].

Hereditary defects collectively referred to as amelogenesis imperfecta (AI) arise from pathogenic variants in genes expressed at specific stages of amelogenesis [25]. X-linked mutations in AMELX affect amelogenin, disrupting the nanosphere scaffold essential for crystal organization [59,142]. Mutations in ENAM, typically inherited in an autosomal dominant pattern, lead to hypoplastic enamel that is thin but relatively well-mineralized [143,144,145]. Loss-of-function variants in MMP20 or KLK4 interfere with protein degradation and clearance, yielding enamel with retained organic content and deficient mineral density [91,146,147,148]. Mutations in genes such as *WD repeat domain 72 (WDR72)*, *SLC24A4*, *and ODAPH* impair vesicle trafficking, ion transport, or ameloblast adhesion, producing hypomaturation phenotypes [102,149,150,151]. In syndromic contexts, enamel defects may occur alongside other ectodermal or metabolic abnormalities, making genetic counseling appropriate [152,153].

Environmental and systemic factors can also compromise enamel development. Excessive systemic fluoride exposure during enamel maturation leads to dental fluorosis, in which altered ion transport, disturbed pH buffering, and impaired proteolysis result in opaque white lines, mottling, and, in severe cases, surface pitting and brown staining [154,155,156]. Molar-incisor hypomineralization (MIH), a common acquired condition, produces sharply demarcated opacities in permanent first molars and incisors, likely due to perinatal hypoxia, systemic illness, or other early childhood stressors affecting ameloblast function during maturation [141,157,158]. Enamel hypoplasia of environmental origin occurs when matrix secretion is prematurely halted by malnutrition, trauma, systemic disease, or localized infection, producing grooves, pits, or thinned enamel surfaces [138,159,160].

Clinically, enamel defects can compromise both function and aesthetics. Hypomineralized enamel is more susceptible to caries due to increased permeability and reduced acid resistance [161]. Exposed dentin beneath hypoplastic or fractured enamel can cause hypersensitivity [162]. Altered surface chemistry, including changes in wettability and mineral composition, can reduce the bond strength of restorative materials. Patients may also experience significant esthetic concerns, particularly when defects affect anterior teeth [163,164].

Management strategies depend on defect severity. Preventive measures include topical fluoride application to enhance surface resistance and the use of desensitizing agents to alleviate hypersensitivity [161]. For localized or moderate defects, resin-based composite restorations or microabrasion may be appropriate [163,165]. Severe generalized defects often require more extensive rehabilitation with porcelain veneers or full-coverage crowns to restore both function and appearance [166,167]. In hereditary cases, early diagnosis facilitates preventive care planning, patient education, and, when indicated, genetic counseling for affected families.

In summary, enamel defects represent the permanent record of developmental disturbances in amelogenesis. Whether arising from genetic mutations, environmental exposures, or systemic insults, these conditions underscore the importance of the molecular and physiological processes described in earlier sections. A detailed understanding of these mechanisms is essential for accurate diagnosis, effective treatment planning, and the development of preventive and regenerative approaches in dentistry.

## 3. Ion Transport During Enamel Maturation

**Figure 2:**
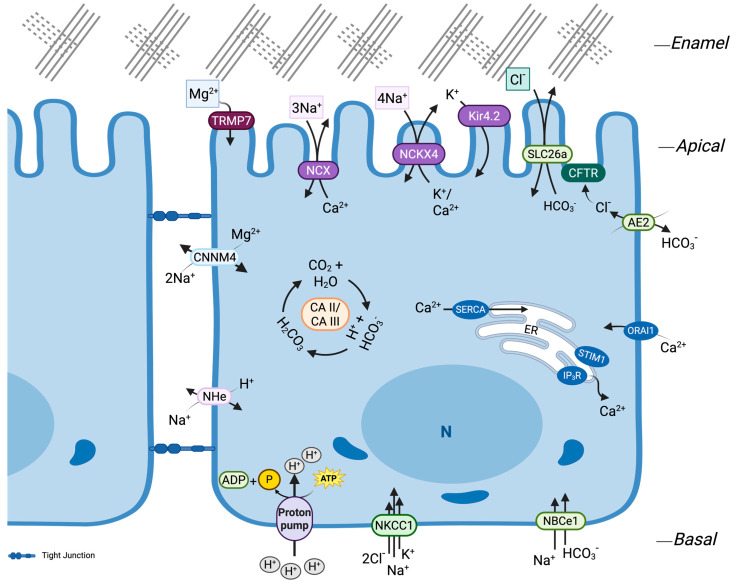
Ion-transport machinery of an ameloblast at the enamel surface. At the basal membrane, NBCe1 loads bicarbonate, while Na^+^-K^+^-2Cl^−^ cotransporter (NKCC1) and the Na^+^/K^+^ pump (not shown explicitly) maintain driving gradients. A V-type H^+^-ATPase (proton pump) provides ATP-dependent proton extrusion. SOCE occurs where ORAI calcium release-activated calcium modulator 1 (ORAI1) in the plasma membrane is activated by Stromal interaction molecule 1 (STIM1) upon ER Ca^2+^ store depletion; ER Sarcoplasmic/endoplasmic reticulum calcium ATPase (SERCA) reloads Ca^2+^, and Inositol 1,4,5-trisphosphate receptor (IP_3_R) releases Ca^2+^ from the ER. Cytosolic carbonic anhydrases (CA II/III) generate H^+^ and HCO_3_^−^ from CO_2_ and H_2_O to fuel pH control. At the apical (enamel-facing) membrane, CFTR provides a Cl^−^ pathway that drives coupled SLC26A exchangers (e.g., A3/A4/A6) to secrete HCO_3_^−^ into the enamel space. AE2 contributes additional Cl^−^/HCO_3_^−^ exchange for intracellular pH balance. Calcium is exported primarily by NCKX4 (1 Ca^2+^ + 1 K^+^ out for 4 Na^+^ in) with auxiliary NCX (3 Na^+^ in:1 Ca^2+^ out). TRPM7 permits Mg^2+^ entry from the matrix, whereas CNNM4 mediates Mg^2+^ efflux (coupled to Na^+^). An Na^+^/H^+^ exchanger (NHE) removes protons produced during crystal growth. Tight junctions between adjacent ameloblasts not only restrict paracellular flux but may similarly influence ion permeability, as shown in other epithelia where passive Ca^2+^ movement occurs through claudin-based channels.

Enamel maturation is the most ion-intensive phase of amelogenesis, during which hydroxyapatite (Hap) crystallites thicken rapidly and must do so within a tightly controlled ionic and acid-base milieu [1,7]. Ameloblasts meet this challenge by adopting a polarized transport program that (i) brings in Ca^2+^ and apically extrudes it in step with crystal growth, (ii) generates and secretes HCO_3_^−^ to neutralize protons, (iii) sustains apical Cl^−^ conductance to keep anion exchange moving, and (iv) uses Na^+^/K^+^ cycling and K^+^ channels to provide the electrochemical work that couples these fluxes (Figure 2) [87,93,168,169,170]. Ruffle-ended ameloblasts (RA) and smooth-ended ameloblasts (SA) modulation synchronizes phases biased toward acid handling, matrix proteolysis, and crystal deposition so that growth proceeds within a narrow chemical window [1,171]. Classic in situ mapping shows alternating acidic (≈pH 5.8–6.0) and near-neutral (≈pH 7.0–7.2) bands under RA and SA cells, respectively, anchoring this functional view in direct pH measurements [172].

### 3.1. Integrated Regulation of Ion Transport During Enamel Maturation

Enamel ion transport is tightly coupled to maturation’s cyclical physiology through stage-dependent expression and membrane polarity. As ameloblasts shift from secretion into maturation, transporters that govern calcium flux, acid-base balance, and monovalent gradients are upregulated and become more sharply polarized [1]. For calcium handling, STIM1, ORAI1, NCX1/3, NCKX4, and plasma-membrane Ca^2+^-ATPases concentrate at their respective domains during maturation, paralleling heightened mineral demand and the alternation between ruffle-ended and smooth-ended forms, where ruffle-ended cells are especially engaged in apical Ca^2+^ extrusion and crystal thickening, and smooth-ended phases favor neutralization and resorption [173]. Dynamic relocalization is also evident in the acid-base machinery. AE2 shifts from apical in secretory cells to lateral or basolateral in maturation, and NBCe1 rises across the secretory-to-maturation transition [87,92]. At the enamel surface, CFTR is essentially confined to the apical pole of maturation-stage ameloblasts, where it assembles with SLC26 exchangers to form a bicarbonate-secreting hub precisely when the proton burden of hydroxyapatite growth is greatest [81,93]. Together, these spatial and temporal patterns underscore a single principle that transporter expression and topology track the surge in mineral accretion and the RA↔SA cycle to maintain a permissive microenvironment for crystal growth and matrix remodeling.

Ion transport supporting enamel maturation is interdependent rather than modular. Basolateral Na^+^/K^+^-ATPase builds the inward Na^+^ gradient and replenishes intracellular K^+^, and supporting layers of the enamel organ supply additional monovalent ions via NKCC1, together establishing the electrochemical and osmotic conditions at the ameloblast interface [174,175,176]. This sodium motive force sustains basolateral NBCe1-mediated bicarbonate uptake and, at the apical pole, powers the K^+^-dependent, Na^+^-coupled NCKX4 exchanger that delivers Ca^2+^ to the mineralization front, making K^+^ recycling a prerequisite for continuous Ca^2+^ export. In turn, Ca^2+^ flux feeds back on matrix chemistry: by shaping the ionic microenvironment at the ruffled border, NCKX4 promotes the acidity that optimizes KLK4 proteolysis, so impaired Ca^2+^ export secondarily reduces protein clearance and undermines maturation [168,177]. Monovalent ions, therefore, sit at the nexus of calcium transport and buffering, while epithelial crosstalk through NKCC1 in adjacent support cells helps maintain a steady Na^+^, K^+^, and Cl^−^ supply to the ameloblast surface throughout the ruffle-ended to smooth-ended cycle [175].

Converging genetic and experimental evidence reinforces this model. Perturbing the SOCE pathway via STIM1 or ORAI1 reduces Ca^2+^ uptake and produces hypomineralized, AI-like enamel, directly validating Calcium release-activated calcium channel (CRAC)-mediated entry in vivo [101,173,178]. Disabling the apical Ca^2+^ efflux arm through SLC24A4 loss abrogates NCKX4 function and yields severe autosomal-recessive amelogenesis imperfecta in mice and humans with protein retention and disorganized crystallites [81,151]. Compromising the bicarbonate axis through Solute carrier family 4 member 4 (SLC4A4) loss causes enamel hypoplasia and hypomineralization, with both local and systemic acid-base disturbances contributing [87,179]. Normalizing systemic pH rescues much of the defect in mice, consistent with NBCe1’s upstream role in base supply [180]. CFTR deficiency produces hypomineralized enamel in mice, with an acidified, chloride- and calcium-poor maturation matrix (pH-indicator positive). *CFTR*-null/ΔF508 pigs likewise show hypomineralized crowns on backscattered-electron imaging, mirroring the higher prevalence of developmental enamel defects reported in cystic fibrosis [169,181,182]. Removal of AE2 disorganizes maturation-stage ameloblasts and produces protein-rich, hypomature enamel, consistent with failed surface pH control [92,93,182]. Beyond divalent and acid-base transport, precise magnesium handling is also essential. CNNM4 loss in Jalili syndrome produces Mg-rich, hypomineralized enamel, and Cnnm4-deficient mice confirm a basolateral Mg^2+^ efflux role for CNNM4 [183,184]. Trpm7 perturbation yields enamel defects that converge mechanistically on reduced alkaline phosphatase activity; in TRPM7-kinase mutants, low ALP activity can be partially restored by Mg^2+^, consistent with the requirement of Mg^2+^ for Tissue-Nonspecific Alkaline Phosphatase (TNAP) catalysis [185]. Finally, systemic disruption of NKCC1 in the surrounding enamel epithelium disorganizes late-maturation ameloblasts and thins enamel despite compensatory increases in other transporters, underscoring that proper gradient generation in support tissues is integral to ameloblast function [175]. Taken together, these phenotypes confirm that enamel maturation depends on a distributed, mutually reinforcing transport network and that defects at any major node predictably collapse the pH cycles, Ca^2+^ delivery, and matrix turnover required for final hardening [1].

### 3.2. Calcium Transport

Calcium transport during enamel maturation is a polarized, stage-tuned process that links basolateral uptake to apical delivery while preserving intracellular homeostasis [1,186]. The prevailing entry pathway is SOCE, in which depletion of endoplasmic reticulum (ER) Ca^2+^ stores, typically following IP_3_ receptor activation, induces oligomerization of the ER sensors STIM1/STIM2 and gating of ORAI1 channels at the plasma membrane (Figure 2) [101,173]. These core SOCE components are robustly expressed in both secretory- and maturation-stage ameloblasts but are upregulated in the maturation stage when Ca^2+^ demand is higher [173]. Their function has been demonstrated in ameloblast systems using thapsigargin to empty ER stores and pharmacologic CRAC blockade to suppress the ensuing Ca^2+^ influx [101,173,178]. Genetic disruption corroborates this mechanism in vivo. Loss of STIM1 or ORAI1 reduces Ca^2+^ entry and produces AI-like enamel defects with disorganized, hypomineralized crowns, underscoring SOCE as the principal route for Ca^2+^ uptake into ameloblasts during both phases of amelogenesis [5,81,178,187].

Once in the cytosol, Ca^2+^ is buffered and partitioned to prevent toxicity while preserving vectorial transport. Cytoplasmic Ca^2+^-binding proteins, including calbindin-D28k, calmodulin, calretinin, and parvalbumin, limit free [Ca^2+^] and facilitate directed transit toward the apical pole [188,189,190,191,192]. In parallel, the ER operates as a dynamic reservoir via SERCA-mediated sequestration and luminal buffering by calreticulin, endoplasmin (HSP90B1), and ERp72. These findings have prompted an ER-assisted “transcytosis” model that complements cytosolic buffering rather than replacing it [1,173,186]. Morphological and physiological evidence support this vectorial handling as electron microscopy shows intracellular Ca^2+^ accumulation preceding its appearance in enamel, and Ussing-chamber measurements demonstrate a temperature-sensitive, basal-to-apical flux consistent with energy-dependent, transepithelial transport [193,194,195].

Apical extrusion of Ca^2+^ into the enamel matrix is mediated by an ensemble of exchangers and pumps. NCKX4 (SLC24A4), a K^+^-dependent Na^+^/Ca^2+^ exchanger, is sharply upregulated during maturation and concentrated at the apical membrane, positioning it as the dominant efflux pathway that supplies Ca^2+^ to the mineralization front. Loss-of-function variants in SLC24A4 cause autosomal-recessive amelogenesis imperfecta in humans and mice, with severely hypomineralized, protein-retentive enamel, highlighting the centrality of NCKX4 to enamel mineral delivery [1,81,168,196]. Additional capacity is provided by NCX1 and NCX3 (SLC8A1/A3), which exchange one Ca^2+^ for three Na^+^ and are localized apically and apicolaterally in both stages, and by plasma-membrane Ca^2+^-ATPases (PMCA; ATP2B1/ATP2B4), which use ATP to extrude Ca^2+^ in exchange for protons and thereby contribute to matrix acid-base balance as well as Ca^2+^ clearance [151,197,198,199,200,201].

Beyond bulk transport, NCKX4 exerts regulatory control over the enamel microenvironment. By shaping local ionic conditions at the ruffled border, NCKX4 promotes the acidity required for KLK4 activity. In its absence, matrix pH rises, KLK4-mediated proteolysis is impaired, and enamel proteins are retained. Restoring acidity rescues KLK4 function in vitro, establishing a mechanistic link between Ca^2+^ efflux and matrix processing [168].

Developmental regulation integrates these elements into the well-known RA to SA ameloblast cycle. Expression and membrane polarization of STIM1, ORAI1, NCKX4, NCX1/3, and PMCA increase during maturation, matching the surge in mineral demand. Ruffle-ended ameloblast phases, dominant during mildly acidic cycles (≈pH 6–6.2), drive apical ion transport and mineral accretion while smooth-ended phases coincide with near-neutral pH (≈pH 7.0–7.2) and favor neutralization, water efflux, and clearance of degraded matrix proteins (KLK4-dependent) [3,54,107]. Together, these data support a model in which SOCE-driven basolateral Ca^2+^ uptake, buffered cytosolic and ER transit, and coordinated apical export form a single, developmentally tuned pathway whose disruption at any node yields the characteristic hypomineralization of enamel pathologies (Table 1) [1,4,101,173,198].

### 3.3. Acid-Base Control: Bicarbonate and Chloride

Acid-base regulation in the enamel organ is a central constraint on mineralization because hydroxyapatite (Hap) crystal growth releases a substantial proton load into the matrix. If not neutralized, this acidity inhibits crystal accretion and protease function [24,107]. Ameloblasts meet this challenge with a polarized bicarbonate-chloride system that is developmentally tuned to the RA to SA cycle and operates in concert with proton pumps and Na^+^/H^+^ exchange to stabilize both extracellular and cytosolic pH (Table 1). The net effect is a tightly choreographed alternation between acidifying phases that favor crystal maturation and neutralizing phases that permit matrix clearance and renewed ion influx [6,87,92,93,168].

At the basolateral membrane, the electrogenic Na^+^/HCO_3_^−^ cotransporter NBCe1 (SLC4A4) provides the principal route for bicarbonate entry (Figure 2). NBCe1 is strongly expressed as cells transition from secretion to maturation and is also detectable in the adjacent papillary layer, positioning the enamel epithelium to draw base from the circulation. Functional relevance is supported by Slc4a4-null models, which develop severe enamel hypoplasia and hypomineralization even without systemic acidosis, and by ameloblast-like cell systems (HAT7) that demonstrate sodium-dependent basolateral-to-apical bicarbonate transport and cytosolic alkalinization when NBCe1 is engaged. Clinical and experimental evidence thus converge on NBCe1 as a key upstream supplier of bicarbonate for enamel buffering [87,177,180,207,219].

Intracellular and extracellular carbonic anhydrases augment this basolateral supply by catalyzing rapid HCO_3_^−^ generation from CO_2_ and H_2_O (Figure 2). Cytosolic CA II and CA III support intracellular production (Table 1). Secreted CA VI operates within the enamel space, and membrane-bound CA XII presents an extracellularly oriented catalytic domain at the ameloblast surface, placing enzyme activity directly at the mineralization front. The spatial deployments of these isoforms, abundant CA II at the apical ends of RA, luminal CA VI bathing the matrix, and CA XII embedded in the apical membrane, create a catalytic scaffold that sustains high-flux buffering precisely where protons are generated. CA XII is a membrane enzyme with an extracellularly oriented catalytic site in epithelia. Car12 mRNA is present in secretory ameloblasts, but definitive protein-level localization at the ameloblast surface is still pending [24,92,94,209,210,250].

Bicarbonate exits apically through a network built around the anion exchanger AE2 and chloride pathways (Table 1). AE2 mediates electroneutral Cl^−^/HCO_3_^−^ exchange and exhibits dynamic polarity: apical in secretory cells and then lateral/basolateral during maturation. Eliminating AE2 in mice produces a characteristic enamel phenotype: retained organic matrix, rapid wear, flattened ameloblasts, and failed modulation between ruffle- and smooth-ended forms, the hallmarks of pH dysregulation in the matrix [10,92,207]. These findings indicate that AE2 functions on both sides of the cell at different stages to maintain transcellular base movement and to keep the extracellular milieu within a narrow pH window compatible with mineral growth [1,88,94,219].

Chloride transport is equally integral, providing both charge balance and a conduit for coupled bicarbonate secretion. CFTR, a cAMP-regulated Cl^−^ channel, is concentrated at the apical pole of maturation-stage ameloblasts and absent from secretory cells, anchoring a stage-specific bicarbonate secretory hub [93]. CFTR assembles multiprotein complexes with SLC26 family exchangers, including SLC26A3, SLC26A4 (pendrin), SLC26A6, and SLC26A7, detected at apical and lateral domains and upregulated during maturation (Table 1). Co-immunoprecipitation demonstrates physical interactions between CFTR and SLC26A6/A7, supporting a model in which CFTR-mediated Cl^−^ flux and SLC26-mediated HCO_3_^−^ exchange operate cooperatively to fine-tune the enamel surface pH during intense mineral deposition [214,251]. In vivo, Cftr-null mice and CFTR-null pigs exhibit under-mineralized, acidified enamel with irregular matrix architecture, paralleling dental manifestations in people with cystic fibrosis and underscoring CFTR’s central role in enamel pH homeostasis [169,182,216].

Additional chloride supply is coordinated across the enamel organ epithelium. Although ameloblasts lack NKCC1, this electroneutral Na^+^-K^+^-2Cl^−^ cotransporter is strongly expressed in the supporting layers (outer enamel epithelium/papillary complex, stellate reticulum, stratum intermedium) from cap through maturation (Figure 2). Loss of NKCC1 disrupts the ameloblast-papillary unit, yielding shorter late-maturation ameloblasts, reduced mineral density, and irregular enamel. In these mutants, NBCe1 and SLC26A3/A6 increase, along with connexin-43 coupling, consistent with a compensatory attempt to sustain ion and fluid flow, yet this is insufficient to normalize enamel formation. Together, NKCC1 in support cells and CFTR at the ameloblast apex establish a directional chloride pathway from the basolateral milieu to the enamel surface, enabling coupled HCO_3_^−^ secretion, sustaining matrix hydration, and coordinating with the ruffle-ended/smooth-ended cycle [68,92,175,182].

Proton handling completes the circuit. Apically, vacuolar H^+^-ATPases are most active in ruffle-ended ameloblasts, where they acidify the enamel space to conditions favoring crystal maturation and matrix proteolysis. Basolaterally, NHE1 exchanges intracellular H^+^ for extracellular Na^+^, stabilizing cytosolic pH and supporting net base secretion when coupled to NBCe1-mediated HCO_3_^−^ entry (Figure 2) [94,171,177]. Ameloblast V-ATPases are compositionally distinct from osteoclast pumps and appear enriched for B1/a4 isoforms during maturation. Notably, a3/TCIRG1 is present at low levels (detection-method dependent), and a3 mutations directly affect ameloblasts and enamel in mice [220]. These mechanisms are embedded within the morphological modulation itself: V-ATPase activity predominates during acidifying ruffle-ended phases, whereas bicarbonate transporters (NBCe1 and SLC26 family) are comparatively prominent during neutralizing smooth-ended phases. Disrupting this alternation by removing AE2 or by chemical stressors such as excess fluoride predictably yields hypomineralized, protein-retentive enamel [171,177,252].

Viewed as a whole, enamel pH control is a polarized, epithelial transport problem solved by coordinated basolateral bicarbonate uptake and cytosolic generation, apical bicarbonate secretion through AE2-CFTR-SLC26 assemblies, targeted proton extrusion, and continuous stabilization of intracellular pH by NHE1 (Table 1). The system’s phase-locked operation with ameloblast morphology ensures that the enamel surface alternates between acidified states that support crystal thickening and neutralized states that allow matrix clearance and renewed ion entry. When any component of this network is compromised, such as NBCe1 or AE2 loss, CFTR deficiency, diminished chloride supply from NKCC1, or stressors that derail modulation, the result is the same: failure to maintain the enamel microenvironment, with consequent hypomineralization and structural fragility [6,92,175,253].

### 3.4. Sodium and Potassium Handling

Sodium and potassium handling in the enamel organ underpins the electrochemical and osmotic landscape that allows ameloblasts to regulate pH, move calcium, and remodel the matrix during maturation. The system is anchored basolaterally by the Na^+^/K^+^-ATPase (NKA), which expels three Na^+^ in exchange for two K^+^ to maintain a steep inward Na^+^ gradient and to replenish intracellular K^+^ (Table 1). This gradient energizes secondary transport processes central to enamel physiology, including bicarbonate import through NBCe1 and apical Ca^2+^ export via the K^+^-dependent Na^+^/Ca^2+^ exchanger NCKX4, thereby linking monovalent ion homeostasis directly to acid-base control and mineral delivery [87,168,174,177,196]. Parallel Na^+^ entry through the basolateral Na^+^/H^+^ exchanger NHE1 supports cytosolic pH stability and sustains the sodium motive force that drives coupled transport. Together, these elements establish the driving forces that make transcellular base movement and Ca^2+^ extrusion possible during the demanding maturation stage [171,186,254].

Although ameloblasts lack notable expression of NKCC1, the Na^+^-K^+^-2Cl^−^ cotransporter is highly expressed in the outer enamel epithelium/papillary complex, stellate reticulum, and stratum intermedium, where it accumulates Na^+^, K^+^, and Cl^−^ to shape the ionic/osmotic milieu at the ameloblast interface [1,175] (Table 1). In mice, Nkcc1 ablation yields shortened late-maturation ameloblasts and thinner, roughened enamel with reduced mineral density. In these mutants, NBCe1, SLC26A3/A6, and Cx43 are upregulated as a partial compensation that nonetheless fails to normalize enamel [68,175]. Thus, support-cell transport supplies chloride and monovalent ions that ameloblasts depend on for pH regulation, fluid balance, and downstream HCO_3_^−^ exchange. Monovalent-driven water flux also cycles with morphology. RA phases favor osmotic inflow (spacing/hydration), SA phases favor reabsorption and protein clearance. Sustained basolateral Na^+^/K^+^ gradients via NKA underpin this maturation-stage choreography (Figure 2) [1,6,170,176].

Potassium homeostasis in the enamel organ is set by NKA at the basolateral membrane, which maintains the inward Na^+^ gradient and replenishes intracellular K^+^ (Figure 2) [170,174]. During maturation, Kir4.2 (KCNJ15) is prominently enriched at the apical border of ruffle-ended ameloblasts, where it supports K^+^ recycling and stabilizes membrane potential (Not shown in the schematic). K^+^-Cl^−^ cotransporters (KCCs) are putative contributors to K^+^ efflux and coupled Cl^−^ movement that could influence matrix hydration, but their definitive localization/function in ameloblasts remains to be confirmed. Critically, intracellular K^+^ availability is functionally coupled to NCKX4. This apical exchanger exports 1 Ca^2+^ together with 1 K^+^ in exchange for 4 Na^+^, making K^+^ recycling/retention prerequisites for sustained Ca^2+^ delivery to the mineralization front. Thus, K^+^ handling is inseparable from the calcium economy of the ameloblast and the maintenance of the ionic microenvironment at the crystal surface [7,151,168,255,256].

These sodium and potassium cycles track the morphological modulation of ameloblasts. During RA phases, Na^+^ absorption and K^+^ efflux predominate, supporting proton secretion, matrix acidification, and proteolysis, while during smooth-ended (SA) phases, K^+^ reabsorption together with NBCe1- and NHE1-mediated Na^+^-bicarbonate and Na^+^-proton exchange drives neutralization and resorptive functions [4,24,168,170,172]. While direct, band-resolved in vivo K^+^ measurements are limited, histology/elemental mapping and transporter localization (e.g., apical Kir4.2 and NCKX4 in RA) corroborate the pattern in which matrix Na^+^/K^+^ generally decline as maturation progresses, with relative K^+^ enrichment during acidic intervals and clearance during neutralizing intervals. This effect becomes especially apparent when modulation or pH regulation is perturbed. The result is a phase-locked monovalent-ion program that coordinates with bicarbonate and calcium pathways to alternate between microenvironments favoring matrix degradation and those conducive to crystal growth and hardening [10,170].

Disruption of any step in this network propagates broadly. Interfering with Na^+^ or K^+^ transport distorts the driving forces for bicarbonate movement and Ca^2+^ export and deranges pH cycles, while loss of NKCC1 in support cells compromises the ionic scaffold needed for normal ameloblast organization and enamel thickness. Conversely, strengthening basolateral Na^+^/K^+^-ATPase-anchored gradients stabilizes downstream exchangers and pumps, preserving the tightly regulated alternation of acidification and neutralization that defines healthy enamel maturation [87,171,175,176].

### 3.5. Magnesium and Trace Elements

Magnesium is a minor yet indispensable determinant of enamel maturation that acts at both cellular and matrix scales. In ameloblasts, magnesium handling is governed by a small cohort of regulators that set intracellular stores and thereby influence crystal nucleation and growth. Chief among these is CNNM4, which is a basolaterally localized, sodium-coupled magnesium efflux transporter (Table 1) [184]. Loss of CNNM4 function causes Jalili syndrome and produces hypomineralized, magnesium-enriched enamel, which directly implicates tight magnesium export in normal mineral development and shows the pathological consequence of intracellular magnesium retention during amelogenesis [183,223]. Additional pathways, such as the magnesium-permeable channel TRPM7, likely contribute to cellular magnesium balance during maturation (Table 1). At the matrix level, magnesium partitions to intergranular phases, notably magnesium-stabilized amorphous calcium phosphate at crystallite boundaries, where it modulates nucleation kinetics and restrains uncontrolled lateral thickening. Together, basolateral magnesium efflux and matrix partitioning make magnesium an essential regulator. When magnesium control fails, enamel remains hypomineralized with altered crystal morphology, whereas proper regulation supports orderly crystal growth and the emergence of hard, resilient enamel [225,227,257].

A complementary axis of Mg^2+^ entry is provided by the divalent cation channel TRPM7, which is strongly expressed in maturation-stage ameloblasts (Figure 2). Deletion of Trpm7 produces an enamel phenotype such as marked hypomineralization with protein retention that closely resembles that of *Alpl*-null mice [225,227,258]. Notably, this phenotype can be partially corrected with dietary magnesium supplementation. Together with the observation that magnesium is a biochemical cofactor for ALPL, these findings support a causal chain in which TRPM7-mediated Mg^2+^ influx sustains ALPL activity at the mineralization front, thereby promoting local phosphate liberation and hydroxyapatite growth [232,259,260].

Elemental analyses place magnesium at about 0.2–0.25% of mature enamel by weight, and classic microprobe work shows a clear increase from the surface toward the DEJ [104,261,262]. This gradient reflects the distinct physicochemical profile of Mg^2+^: its smaller ionic radius and higher hydration energy reduce its compatibility with the hydroxyapatite lattice and favor localization at crystal interfaces rather than deep substitutional sites [263,264]. In consequence, Mg^2+^ tends to accumulate at crystal boundaries, where it modulates surface energy and growth kinetics and thereby influences enamel solubility and the propensity for post-eruptive acid attack [104,257].

Beyond steady-state distribution, magnesium participates in the transient mineral phases that precede ordered crystal formation. Mg-substituted amorphous calcium phosphate (Mg-ACP) has been detected in early enamel and along crystal boundaries, consistent with a role in stabilizing amorphous precursors, governing the amorphous-to-crystalline transition, and shaping crystallite habit [257,264,265]. The same interfacial chemistry likely underlies Mg-ACP’s reported contributions to fluoride uptake and lesion repair dynamics during early remineralization, linking a trace ion to clinically relevant resilience of the enamel surface [266,267].

These cellular and matrix-level roles position magnesium as both an enzymatic cofactor and a microstructural regulator during maturation. CNNM4-driven efflux prevents pathological Mg^2+^ accumulation in ameloblasts, while TRPM7-dependent influx supplies the divalent cation needed to optimize ALPL function. At the same time, the intrinsic chemistry of Mg^2+^ biases it toward crystal surfaces and amorphous intermediates, where it fine-tunes nucleation and growth. When any element of this circuitry is perturbed, such as reduced TRPM7 activity, defective CNNM4 efflux, or altered Mg^2+^ availability, the consequences cascade from impaired phosphatase activity to disordered mineral assembly, culminating in the characteristic hypomineralized, protein-retentive enamel seen in genetic models and human disease [183,184,227].

Although magnesium is the best defined among the trace elements in enamel, iron provides an instructive counterpoint. In rodents, ameloblasts deposit an iron-rich layer late in maturation, mostly prominent in continuously erupting incisors, that produces the characteristic surface pigmentation [242,268]. This outer iron-rich enamel is measurably more acid-resistant and wear-tolerant than underlying enamel, a conclusion supported by classic etch tests and by recent high-resolution analyses of the iron-enriched surface [269,270,271,272]. Maturation-stage ameloblasts upregulate ferritin heavy chain, reflecting a need for safe intracellular iron sequestration to limit oxidative stress and to support elevated mitochondrial oxidative phosphorylation during the high-demand phases of ion transport and matrix remodeling [1,241,242,270,273,274]. These observations suggest a terminal role for iron in refining surface properties once calcium and phosphate loading is largely complete, and they underscore the broader principle that low-abundance ions can exert outsized influence on enamel’s structure and function.

### 3.6. Citrate Transport and Its Role in Enamel Mineralization

Beyond divalent cation regulation, citrate handling has emerged as a contributor to enamel mineral chemistry. The Na^+^-coupled citrate transporter SLC13A5 (NaCT) mediates electrogenic citrate influx (≈ 4 Na^+^:1 citrate^3−^) and links epithelial ion transport to intermediary metabolism, positioning citrate to influence HAp growth through transient Ca^2+^ chelation and surface charge modulation [230]. Genetic loss of Slc13a5 in mice produces failure of enamel maturation with aberrant matrix and markedly reduced mineralized enamel, consistent with an AI-like phenotype, indicating that citrate supply is functionally relevant to amelogenesis [229]. Evidence from metabolic studies further supports a role for NaCT-dependent citrate partitioning in mineralizing tissues, with tooth defects reported when this pathway is perturbed [275].

With respect to cellular localization, recent work localizes NaCT to ameloblast membranes (including basolateral domains and the papillary layer), and sensitive reporter models suggest broad membrane distribution that could support context-dependent citrate flux at the enamel organ interface [231,275]. At the crystal level, citrate adsorbs to apatite surfaces and stabilizes amorphous calcium phosphate (ACP) precursors by chelating Ca^2+^, delaying premature crystallization until local ionic composition and pH favor HAp formation, mechanistically aligning citrate transport with the RA↔SA cycles that tune matrix chemistry [276,277].

### 3.7. Phosphate Transport and Regulation During Enamel Maturation

Inorganic phosphate (Pi) is indispensable for the nucleation, elongation, and stabilization of hydroxyapatite in enamel, and its supply increases during maturation as crystals expand in width and thickness [6]. Because crystal growth releases protons into the extracellular space, phosphate flux must be synchronized with calcium delivery and acid-base control. A useful net expression highlighting this proton burden is: 10 Ca^2+^ + 6 HPO_4_^2−^ + 2 OH^−^ → Ca_10_(PO_4_)_6_(OH)_2_ + 6 H^+^, that is consistent with the well-established observation that hydroxyapatite precipitation acidifies the enamel fluid and necessitates bicarbonate buffering (Figure 2) [24]. These stoichiometric constraints explain why phosphate handling is tightly coupled to ameloblast modulation, sodium-driven transport, and extracellular pH during maturation, precisely when mineral content and crystallite organization rise most steeply [1,6,87].

#### 3.7.1. Historical Insights into Phosphate Dynamics

During maturation, phosphate delivery to the enamel surface occurs in discrete pulses rather than as a steady flux. Whole-mount radioautography shows that within ~5 min of an intravenous ^33^P-orthophosphate pulse, label appears as sharply banded uptake confined to the maturation zone and aligned with ruffle-ended/smooth-ended (RA/SA) ameloblast bands [278], an organization independently tracked by polychrome labeling and GBHA staining of sequential SA waves [279]. Compositional contrast by backscattered-electron imaging corroborates the radioautographic band/interband pattern, reinforcing the view that mineral addition is spatially periodic across the zone [280]. Classic tracer work with ^32^P further indicates that early (pre-maturation) enamel rapidly exchanges phosphate and loses a substantial fraction of label on cold chase, whereas maturation-stage enamel retains most of the label, consistent with durable crystal incorporation at this stage [281]. Chemical profiling across the rat incisor likewise documents stage-dependent shifts in composition that parallel this radiotracer behavior [282]. These pulses coincide with extracellular pH oscillations recorded as alternating acidic and near-neutral bands on developing enamel and with ameloblast-driven cycles of proton secretion and buffering during maturation, providing a mechanistic basis for the coupling of phosphate influx to Ca^2+^ export and pH control as crystals thicken [6,172].

#### 3.7.2. Phosphate Transporters

**Figure 3:**
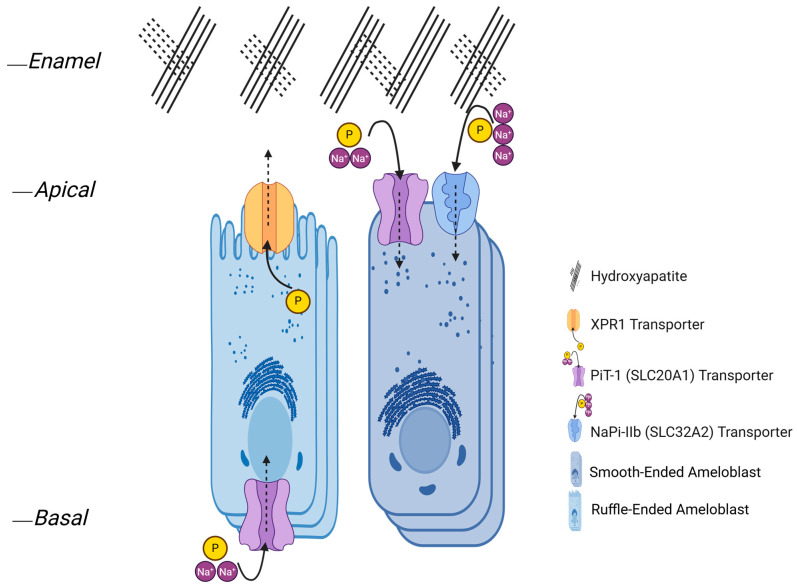
Schematic representation of phosphate transporters in ameloblasts during the maturation stage of amelogenesis. During the maturation stage, ameloblasts alternate between ruffle-ended and smooth-ended morphologies, both of which are illustrated here. To avoid overcrowding, two transporters are shown on each cell rather than all four on a single ameloblast. The main phosphate transporters implicated in ameloblast function are highlighted. PiT1 on the basolateral side mediates sodium-dependent phosphate uptake from the blood/interstitial fluid, while NaPi-IIb at the apical side imports phosphate from the enamel matrix and XPR1 exports phosphate toward the enamel space. The number and type of ions transported by each channel are indicated in the schematic. Hydroxyapatite crystals in the enamel matrix are shown at the apical surface to indicate the site of mineralization.

Multiple transporter families contribute to phosphate movement across ameloblasts. PiT1/SLC20A1 and PiT2/SLC20A2, and the NaPi-IIb (SLC34A2) mediate cellular uptake, with stage-responsive expression in the enamel organ and known pH sensitivity from their biophysics and epithelial roles (Figure 3) (Table 1).

In mice, NaPi-IIb mRNA is negligible in secretory ameloblasts but rises sharply in maturation, whereas PiT1 is robustly expressed in ameloblasts across secretory and maturation stages, with little signal in odontoblasts, supporting a primary role in ameloblast Pi uptake. PiT2 shows a developmentally dynamic pattern that is a transient, strong signal in young secretory ameloblasts, followed by predominant expression in adjacent enamel-organ support layers (stratum intermedium/papillary; sub-odontoblastic layer) at later stages [1,9,10]. Functionally, both SLC20 carriers prefer H_2_PO_4_^−^ over HPO_4_^2−^ ions operating across acidic-alkaline ranges and use a 2 Na^+^:1 Pi stoichiometry, so their apparent kinetics vary with extracellular pH via phosphate speciation (H_2_PO_4_^−^↔HPO_4_^2−^) rather than a strict pH switch. NaPi-IIb exhibits an optimal activity near mildly acidic pH with regulation by systemic acid-base status (Table 1) [240,283,284]. On the efflux side, the eukaryotic exporter XPR1 is strongly implicated as the apical route for phosphate delivery to the enamel space. Xpr1 is expressed in developing teeth alongside Pi importers, and recent structural work defines XPR1 as the dedicated transmembrane Pi exporter in mammalian cells (Table 1) [9,11]. Their detailed roles, polarity, and regulation will be addressed in the next subsection.

Phosphate delivery is burst-like and synchronized with RA/SA cycling and pH control, supported by intracellular stores and phosphatase activity, and bounded by systemic phosphate availability [6,172,278]. Recent transcriptomic, in situ hybridization, and immunohistochemical studies have clarified the machinery that mediates Pi flux across the enamel organ. Three systems account for most of the major traffic during amelogenesis: type III SLC20 symporters, the type II NaPi-IIb, and the eukaryotic exporter XPR1 [1,5,9,285]. Their expression is stage-specific and epithelial-domain specific within the enamel organ, tracking functional shifts from secretion to maturation and responding to changes in the extracellular milieu, including pH. Bulk and cell-type transcriptomics and in situ hybridization localize Slc34a2 and Slc20a1 to ameloblasts, with Slc34a2 showing a pronounced upregulation in maturation, while Xpr1 is co-expressed at lower levels. This depicts an arrangement consistent with burst delivery of Pi to the mineralization front during RA phases [9,29,80].

(a) SLC20 family: PiT1 (SLC20A1) and PiT2 (SLC20A2)
  PiT1 and PiT2 are type III Na^+^-phosphate symporters that import the monovalent phosphate species (H_2_PO_4_^−^) with an electrogenic 2 Na^+^:1 Pi stoichiometry, a property established by heterologous transport measurements and refined by recent structural work (Table 1) [237,286]. In vivo mapping across murine tooth germs shows Slc20a1/PiT1 is predominantly expressed in ameloblasts, with signal strongest postnatally and most evident in maturing cells, while odontoblasts are largely negative in these sections. By contrast, multiple dentin/odontoblast model systems and human pulp-derived odontoblasts do express SLC20A1 in vitro, indicating species-, stage-, and model-dependent differences. PiT2 shows a developmentally dynamic pattern that includes a transient but strong signal that appears in secretory ameloblasts, while high and persistent expression is found in the stratum intermedium and, later, the papillary and sub-odontoblastic layers as teeth mature [9,287]. More recent systematic in situ/LacZ analyses similarly localize Slc20a2 away from ameloblasts and into supporting layers [9,287]. Functionally, the SLC20 carriers are widely regarded as “housekeeping” phosphate importers that maintain intracellular Pi for ATP generation and biosynthesis in polarized epithelia. In enamel organs, this role aligns with the energy-intensive transitions from secretion into maturation [288,289]. Older foundational studies that first identified the PiT family as Na^+^-dependent phosphate symporters using viral receptor clones in oocytes remain key precedents for their transport identity [290].
(b) SLC34 family: NaPi-IIb (SLC34A2)
  NaPi-IIb is the type II sodium-phosphate cotransporter identified in the enamel organ and operates as an electrogenic 3 Na^+^:1 HPO_4_^2−^ carrier, providing high-capacity Pi transport (Table 1) [237,291]. In rodents, NaPi-IIb expression is low in secretory ameloblasts and rises sharply in maturation, and immunolocalization shows intense signal over the apical plasma membrane of early and late maturation ameloblasts with only weak apical staining in secretory cells. Papillary cells also stain for NaPi-IIb. This stage- and domain-specific pattern aligns with the increased mineral demand and RA↔SA modulation that characterize maturation [1,10]. Beyond stage control, NaPi-IIb function and abundance are pH- and milieu-responsive in epithelia. In the intestine, it exhibits pH-dependent transport kinetics, and its brush-border abundance increases during metabolic acidosis, suggesting a general capacity for acid-linked up-regulation, although this has not yet been demonstrated directly in ameloblasts [292,293,294]. Reports of strong apical NaPi-IIb in late maturation indicate a potential role in the apical Pi uptake from the enamel space into the ameloblasts (Figure 3). Any apical efflux toward the matrix would require a bona fide exporter such as XPR1, but such an apical efflux role remains highly speculative for NaPi-IIb [10].
(c) XPR1 (phosphate exporter)
  XPR1 is the only recognized inorganic phosphate exporter in mammalian cells, and structural and biochemical work now defines its Pi-export mechanism [11,295]. In teeth, XPR1 is expressed during postnatal stages when enamel mineralization accelerates, rising alongside other Pi transporters as ameloblasts shift from matrix secretion toward protein resorption and crystal deposition (Figure 3); this timing is compatible with an efflux role at the matrix-facing surface, although direct membrane-polarity mapping in ameloblasts remains limited. Taken together, the convergence of export mechanism (from other tissues) and developmental expression (in enamel organs) makes XPR1 the leading candidate for the apical efflux limb that complements SLC20/SLC34-mediated uptake (Table 1) [9,11].

Viewed as a system, PiT1, PiT2, NaPi-IIb, and XPR1 form a vectorial, temporally coordinated pathway that couples basolateral Na^+^-driven Pi uptake to controlled delivery at the distal (matrix-facing) surface (Figure 3). Expression studies in mouse and human tooth germs localize Slc34a2 and Slc20a1 to ameloblasts (with Slc20a2 enriched in supporting layers), and document higher postnatal abundance of XPR1, supporting stage-specific roles across secretion, transition, and maturation. During the secretory stage of enamel formation, Pi entry is dominated by PiT1 and NaPi-IIb, whereas maturation increasingly relies on regulated efflux via XPR1 to fine-tune extracellular Pi, where crystals thicken and widen [9,11]. Stage-dependent changes in transporter abundance and polarity synchronize phosphate supply with Ca^2+^ export and acid-base control across RA and SA cycles, ensuring precise crystal accretion [29,107]. Finally, pH-responsive regulation of NaPi-IIb is well-documented in epithelia such as the intestine, where acidosis increases NaPi-IIb abundance and transport. This provides a mechanistic precedent for acid-linked up-regulation during the acidic phases of ameloblast modulation, although the direct pH-challenge data in enamel are still emerging [294,296].

#### 3.7.3. Transport Mechanisms and Intracellular Handling of Phosphate During Enamel Maturation

The intracellular fate of phosphate (Pi) in ameloblasts remains incompletely defined, but evidence from enamel and skeletal systems indicates that Pi handling is dynamic, compartmentalized, and enzyme-coupled rather than a simple transcellular conduit. Forming enamel passes through an ACP stage before crystallization, implying regulated precursor control within the epithelium [265,297]. Similarly, mineralizing osteoblasts harbor phosphate- and calcium-rich vesicles contiguous with mitochondria that contribute directly to matrix mineralization [265,298]. After entry, Pi is likely sequestered, chemically modified, and released in a temporally controlled manner, consistent with mammalian evidence for polyphosphate (polyP) pools in mitochondria and in acidic, acidocalcisome-like vesicles [299,300,301].

In a working model extrapolated from mineralizing cells, a portion of imported Pi is condensed into polyP, linear chains of orthophosphate linked by high-energy phosphoanhydride bonds, within mitochondria or acidocalcisome-like compartments [299,302]. PolyP possesses properties well-suited to regulate calcium-phosphate systems. It chelates Ca^2+^ and interferes with apatite nucleation in a chain-length- and concentration-dependent fashion as it is hydrolytically labile under acidic conditions, and it can associate with and stabilize ACP-like precursors [303]. In ameloblasts, these properties could provide dual functionality. Buffering intracellular phosphate as a mobilizable reserve while stabilizing precursor phases to prevent premature hydroxyapatite formation until ionic composition and pH become favorable. This concept aligns with ACP-to-apatite transitions in enamel and demonstrates that polyP-stabilized ACP converts to apatite under mineralizing conditions [265,304].

At the physicochemical interface, polyP adsorption to nascent mineral surfaces slows phase-transformation kinetics and delays premature crystallization, effects shown historically on hydroxyapatite particles and surfaces [305,306]. Such tunability maps onto enamel’s RA/SA cycling, wherein the extracellular milieu oscillates between mildly acidic and near-neutral states during maturation.

Enzymatic processing at the enamel surface provides the complementary switch from inhibition to supply. ALPL is strongly expressed in the enamel organ and becomes enriched at the distal (apical) pole of transition- and maturation-stage ameloblasts, positioning catalytic activity at the mineralization front [50,307]. In this location, ALPL can hydrolyze polyP to orthophosphate and degrade pyrophosphate (PPi), a potent inhibitor of hydroxyapatite formation [308,309,310]. Because ALPL activity depends on local pH and divalent cofactors such as Mg^2+^ and Zn^2+^, apical confinement affords spatial control over extracellular Pi generation and helps avoid cytosolic Pi overload or nonspecific precipitation [311]. This arrangement parallels TNAP’s established role at skeletal mineralization fronts, where matrix-vesicle-rich microenvironments require local PPi hydrolysis and Pi provision to initiate and propagate mineral [312,313]. Notably, alkaline phosphatase isoforms differ in polyP substrate preference: intestinal alkaline phosphatase acts as a highly active exopolyphosphatase across a broad range of chain lengths, whereas ALPL shows limited or context-dependent activity toward longer polyP and reduced activity when polyP is adsorbed to apatite [308,309,314]. These biochemical constraints likely shape how efficiently polyP stores are converted to Pi at the crystal interface.

A vesicular route that couples polyP storage to apical hydrolysis is plausible in ameloblasts, even though no dedicated vesicular Pi transporter has been identified. Current expression maps list plasma-membrane Pi importers (SLC20A1/2; SLC34A2) and the exporter XPR1, leaving the intravesicular conduit undefined [9]. Nonetheless, mammalian cells contain mitochondrial polyP and polyP-containing, acidocalcisome-like vesicles, and mineralizing tissues deploy matrix-vesicle pathways that initiate mineral seeding; osteoblast-derived vesicles concentrate polyP, Ca^2+^, and phosphatases to nucleate mineral within lipid-bounded microenvironments [312,315,316,317,318,319]. By extension, ameloblasts may route polyP-bearing cargo to the distal membrane, releasing polymer that is then hydrolyzed by apical ALPL to generate short, burst-like increases in Pi coincident with ruffle-ended phases, when mineral growth and acid production peak [1,302,315,320]. Dysregulation of polyP turnover, impaired enzymatic degradation, or defects in vesicular trafficking/transporter coupling would therefore be expected to yield hypomineralization phenotypes, as seen when polyP pathways are perturbed in other mineralizing systems [302,314,321].

Membrane transporters set the intracellular availability and polarity of Pi movement that feed this vesicular-enzymatic axis. Basolateral uptake via Na^+^/Pi cotransporters of the SLC20 and SLC34 families likely establishes the cellular Pi pool in a stage-dependent manner [9]. A key nuance is the polarity of NaPi-IIb. Although canonically an importer, NaPi-IIb shows strong apical localization in late-maturation ameloblasts [10].

Given well-documented pH-linked regulation of NaPi-IIb in intestinal epithelium, enamel-specific ionic conditions, and potential coupling to vesicular cycling, apical NaPi-IIb could still contribute to distal Pi handling without invoking true efflux [294]. Any bona fide inorganic phosphate efflux across the plasma membrane would most plausibly require XPR1, the only recognized mammalian Pi exporter, whose mechanism has now been defined structurally, and XPR1 is co-expressed with SLC20/34 transporters during postnatal enamel development, consistent with roles at stages when mineral deposition accelerates [9,11]. Thus, while unproven in ameloblasts, the combination of apical NaPi-IIb expression (as an apical importer in maturation) and pH-sensitive regulation keeps it among the candidates that could act alongside XPR1 and apical enzymatic pathways at the matrix-facing pole [10,294].

Taken together, current data support a cohesive, testable model in which basolateral SLC20/SLC34-mediated uptake establishes intracellular phosphate availability; polyP synthesis and vesicular trafficking provide temporal buffering and spatial targeting of phosphate cargo; and apically enriched ALPL converts stored polyP and PPi into orthophosphate precisely at the mineralization front, thereby both supplying substrate and removing inhibition [9,302,307,310]. Priority experiments include identifying polyP-handling enzymes in ameloblasts, verifying the presence and routing of polyP-rich vesicles, and determining whether ALPL functionally interfaces with Na^+^/Pi transporters and XPR1 to coordinate mineral accretion and pH cycles at maturation [312,315,316].

#### 3.7.4. Systemic Versus Local Regulation of Phosphate Availability (With Temporal Dynamics of Incorporation)

Although enamel mineralization occurs within the enamel organ, it is ultimately constrained by whole-body phosphate (Pi) balance. The kidney reclaims most filtered Pi, and the intestine supplies dietary Pi; therefore, circulating Pi sets the ceiling for enamel use. In mammals, the dominant renal apical transporters are type II Na^+^/Pi cotransporters NaPi-IIa (SLC34A1) and NaPi-IIc (SLC34A3) in proximal tubule brush border, and loss-of-function in either gene causes renal Pi wasting and hypophosphatemic disease in humans (e.g., SLC34A3-related HHRH; SLC34A1-related proximal tubular Pi-leak phenotypes) [322,323,324]. By contrast, these renal isoforms are essentially absent from developing teeth. In mouse and human tooth germs, SLC34A1 and SLC34A3 are very low/undetectable, whereas SLC34A2 (NaPi-IIb), SLC20A1/2 (PiT1/2), and the exporter XPR1 show robust enamel-organ expression, especially postnatally [9].

Locally, enamel-organ transporters meter delivery at the mineralization front. As detailed above, NaPi-IIb is polarized to the distal domain in maturation-stage ameloblasts; here we note the quantitative shift that accompanies this polarity. NaPi-IIb transcripts rise dramatically across the secretory to maturation switch (≈64-fold in rodent ameloblastomics) [10,29]. Ameloblast modulation imposes alternating acidic and near-neutral matrix bands that Pi delivery must track; classic indicator-dye work recorded ~pH 5.8–6.0 versus ~7.0–7.2 stripes during maturation [6,172]. While direct pH-dependent control of *SLC34A2* in ameloblasts remains unproven, epithelial precedents show NaPi-IIb protein can be upregulated several-fold by metabolic acidosis in vivo, consistent with enamel’s acid-base oscillations [294]. In this systemic-local framework, the kidney + intestine set substrate availability; the enamel-organ transporters determine where or when that substrate is immobilized.

The dependence on systemic Pi becomes clinically evident when whole-body homeostasis is perturbed. Inherited or acquired hypophosphatemia, most prominently X-linked hypophosphatemia (XLH) and renal tubular disorders such as Fanconi syndrome, limits Pi delivery to mineralizing tissues and yields hypoplastic or hypomineralized enamel [325,326,327]. Mouse models disrupting renal Na^+^/Pi cotransporters (NaPi-IIa or NaPi-IIb) exhibit systemic Pi wasting and skeletal mineralization defects, providing a mechanistic bridge to enamel pathology under hypophosphatemic conditions, although enamel phenotypes per se are less completely characterized in these specific knockouts [328]. Notably, the absence of SLC34A1/3 from ameloblasts implies that enamel defects in hypophosphatemic states arise secondarily from reduced circulating Pi rather than loss of a local enamel isoform [9]. Because enamel is acellular and non-remodeling after eruption, any mismatch between circulating Pi and ameloblast transporter capacity during development leaves irreversible defects [1,86].

Temporal radiotracer analyses refine this model by pinpointing when and where Pi is incorporated. Pulse-chase ^32^P studies reveal a biphasic, developmentally regulated process that demonstrates an early, labile secretory-stage phase marked by rapid uptake into superficial regions that is highly exchangeable (on cold-Pi chase, ~40% label loss, indicating transient binding and high matrix water accessibility), and a prolonged maturation phase in which Pi becomes stably incorporated into hydroxyapatite. The maturation zone retains most label (~10% loss), consistent with lattice entrapment as crystals thicken and porosity declines [6]. In vivo ^33^P radioautography shows sharply banded incorporation confined to the maturation zone that aligns with RA/SA modulation, underscoring pulsatile, spatially restricted Pi delivery rather than steady accretion [270]. In vitro complements agree: initial labeling concentrates just proximal to the “opaque” boundary marking the onset of maturation, then broadens and homogenizes as diffusion equilibrates and surface area declines with progressive mineralization [274,327]. Shortly after ^33^P administration (~10 min-4 h), two specific-activity peaks emerge, one in early forming enamel and another at the maturation onset, and over time the dominant peak shifts distally with the eruption trajectory of rat mandibular incisors (≈0.6 mm/day under impeded conditions), confirming that phosphate incorporation couples to tissue movement and ameloblast progression [4,6,329,330]. Concordant pH-indicator stripes (acidic ~5.8–6.0 vs. near-neutral ~7.0–7.2) tie these phosphate pulses to coordinated bicarbonate secretion and matrix processing during the transition from amorphous precursors to organized hydroxyapatite [172,278].

Together, these observations support a temporospatial scheme in which systemic mechanisms maintain circulating Pi required for enamel formation, while local, pH-sensitive transporter programs and ameloblast modulation determine when and where that phosphate is immobilized in the lattice during maturation. This dual-layered regulation confers robustness but also defines vulnerabilities such as dietary insufficiency, genetic disruption of systemic Pi handling, or perturbation of local transporter regulation that can derail delivery during critical windows and leave permanent defects in non-remodeling enamel [6,24].

## 4. Conclusions and Future Perspectives

Enamel formation is a temporally ordered integration of matrix turnover, epithelial ion transport, and extracellular acid–base control, and maturation succeeds only when these programs advance in phase. The evidence surveyed here across developmental staging, transport physiology, and clinical genetics supports a view in which tissue durability arises from the synchronization of cellular modulation with interfacial chemistry.

Within this integrated picture, phosphate transport is a principal organizing axis of maturation. Classical observations of radiotracer banding and surface pH oscillations indicate episodic and spatially delimited phosphate delivery that aligns with ameloblast modulation. Crystal thickening proceeds efficiently only when phosphate availability, interfacial pH, and matrix proteolysis are co-timed, and perturbations that alter the amplitude or timing of any one of these processes predictably impair mineral accretion and leave enamel enriched in protein and water.

A concise mechanistic framework follows. Basolateral uptake of phosphate, intracellular buffering that separates uptake from release in time, controlled apical export into the forming tissue, and enzymatic hydrolysis at the mineralization front that removes inhibitors such as pyrophosphate collectively sustain orthophosphate activity at the crystal surface. This flux is functionally coupled to bicarbonate-based proton handling and to calcium delivery, so the effective balance among Ca, Pi, and pH at the interface is set by epithelial transport rather than by bulk fluids. The specific molecular details are developed in the main text, and the key point here is that coordination among modules rather than the maximal capacity of any single module governs maturation outcomes.

Resolving the remaining uncertainties requires quantitative experiments in situ. Transporter topology and dynamics must be mapped across ruffle-ended and smooth-ended phases. Phosphate buffering and release at the ameloblast interface need molecular specificity. The phase relationships among phosphate delivery, acid–base compensation, calcium flux, and proteolysis should be measured within the same preparation. Spatially registered molecular atlases along the incisor growth axis, live reporters for key ions integrated with ultrastructural readouts, and targeted inducible perturbations provide a tractable path to causal resolution and to parameterized models that predict crystal thickening and tissue mechanics.

Clinical translation should treat phosphate delivery as a controllable variable. Systemic correction of hypophosphatemia should be evaluated with enamel-specific endpoints that report crystal thickness, residual matrix and water, and fracture behavior, but not hardness alone. Local and time-restricted enhancement of phosphate availability or interfacial enzymatic activity during maturation merits testing, provided that acid–base and calcium modules are co-optimized to avoid ectopic or heterogeneous precipitation. Biomimetic repair strategies that tune the interfacial balance among Ca, Pi, and pH and that leverage condensed phosphate chemistries may standardize the quality of enamel-like mineral produced ex vivo and inform minimally invasive interventions. Incorporating genotypes that affect phosphate handling into care pathways can improve risk stratification and the timing of definitive restorations.

Taken together, the synthesis advanced here reframes amelogenesis as a phosphate-paced physiology that coordinates epithelial transport with matrix physics to produce a durable and near anhydrous ceramic. Centering phosphate as the integrator moves the field from descriptive staging toward predictive and testable models and toward mechanism-guided strategies for prevention, diagnosis, and repair.

## Figures and Tables

**Table 1:** Ion Transporters and Regulators in Amelogenesis.

Ion(s)	Gene/Protein	Ions Transported	Function in Ameloblasts	Location	Stoichiometry	Disease Associations	Key References
Ca^2+^	*STIM1/STIM2* + *ORAI1* (SOCE/CRAC)	Ca^2+^ influx (store-operated)	Basolateral SOCE that replenishes cytosolic and ER Ca^2+^ to support maturation-stage transport and apical efflux	ORAI1 at basolateral plasma membrane; STIM1/2 in ER	Channel-mediated, non-stoichiometric	Loss/impairment → hypomineralized, AI-like enamel (Stim1/2 cKO; ORAI perturbation); STIM1 mutations linked to AI in humans	[101,173,186,202]
Ca^2+^	*ATP2A* (SERCA)	Ca^2+^ into ER (H^+^ countertransport)	Refills ER Ca^2+^ stores; buffers cytosolic Ca^2+^ and terminates SOCE	ER membranes	2 Ca^2+^ in:2–3 H^+^ out per ATP	ATP2A2 → Darier disease (skin); General Ca^2+^ imbalance; not AI-specific	[101,173,203,204,205]
Ca^2+^	*SLC24A4* (NCKX4)	Ca^2+^ efflux, Na^+^ in, K^+^ out	Major Ca^2+^ exporter during maturation; supports matrix mineralization and optimizes KLK4-mediated protein clearance	Apical membrane of ruffle-ended ameloblasts	Electrogenic 4 Na^+^ in:1 Ca^2+^ + 1 K^+^ out	Biallelic SLC24A4 variants → autosomal-recessive hypomaturation AI	[81,101,151,168,196,201]
Ca^2+^	*SLC8A1/3* (NCX1/3)	Ca^2+^ efflux, Na^+^ influx	Supplementary Ca^2+^ extrusion during maturation (minor vs. NCKX4)	Apical (Tomes’ process); remains detectable during the maturation stage	3 Na^+^ in:1 Ca^2+^ out (electrogenic)	Not directly linked to AI	[101,196,197,198,206]
Ca^2+^	*ATP2B1/4* (PMCA1/4)	Ca^2+^ efflux, H^+^ counter-transport	ATP-driven clearance of cytosolic Ca^2+^; supports acid-base balance; complements NCKX4 during maturation	Basolateral, with occasional reports of apical/apicolateral labeling	1 Ca^2+^ out:2 H^+^ in per ATP (overall electroneutral)	No direct AI; perturbation associates with hypomineralized enamel (e.g., Atp2b1a knockdown impairs tooth mineralization; PMCA4 reduced in Mmp20^−^/^−^ enamel)	[198,199]
HCO_3_^−^ (with Na^+^)	*SLC4A4* (NBCe1)	Na^+^ + HCO_3_^−^ influx	Basolateral bicarbonate supply	Basolateral	1 Na^+^ + 2–3 HCO_3_^−^ in	Mutations → enamel hypoplasia	[87,179,180,207,208]
HCO_3_^−^/H^+^	Carbonic anhydrases: *CA2/CA6/CA12* (CA II/VI/XII)	CO_2_ hydration → H^+^ + HCO_3_^−^	Local HCO_3_^−^ generation to support apical HCO_3_^−^ secretion and matrix pH control during maturation	CA II cytosol (ameloblasts and papillary layer); CA VI secreted → enamel fluid; CA XII membrane/apical	Enzymatic (reversible; no fixed stoichiometry)	CA II deficiency → enamel defects/hypoplasia	[24,209,210,211]
HCO_3_^−^/Cl^−^	*SLC4A2* (AE2)	Cl^−^/HCO_3_^−^ exchange	Basolateral HCO_3_^−^ extrusion and Cl^−^ uptake to support pH homeostasis and apical HCO_3_^−^ secretion	Apical (secretory)/basolateral (maturation)	1:1 (electroneutral)	Slc4a2/Ae2 knockout → hypomineralized enamel, failed maturation	[92,207,212]
HCO_3_^−^/Cl^−^	*CFTR* (CFTR)	Cl^−^ and HCO_3_^−^ (HCO_3_^−^-permeable anion channel)	Apical anion conductance that permits Cl^−^ efflux and supports HCO_3_^−^ secretion to neutralize protons during maturation; provides Cl^−^ recycling for apical Cl^−^/HCO_3_^−^ exchangers (e.g., SLC26 family)	Apical membrane of maturation-stage ameloblasts	Channel; electrodiffusive (no fixed stoichiometry)	Loss-of-function (cystic fibrosis) → hypomineralized, acidic maturation bands; altered Cl^−^/Ca^2+^ content in Cftr-null enamel	[1,169,177,213]
HCO_3_^−^/Cl^−^	*SLC26A1* (Sat1)*/A3* (Dra)*/A4* (pendrin)/A6 (Pat1)*/A7* (Sut2)	Cl^−^/HCO_3_^−^ exchange (CFTR-coupled)	Apical HCO_3_^−^ secretion for matrix neutralization and protein clearance (maturation)	Apical (A1, A3, A4 and A6); apical and supranuclear (A4); apical, partly subapical, cytoplasmic (A7)	Mostly 1:1; A6 often 1 Cl^−^:2 HCO_3_^−^	No direct enamel disorder; A4→Pendred (systemic); Slc26a7 deletion delays enamel (rat)	[214,215,216,217]
H^+^	*ATP6V* (V-ATPase; e.g., ATP6V1B1/ATP6V0A4)	H^+^ efflux (proton pump)	Apical acidification during ruffle-ended phases; endo-lysosomal acidification supporting matrix protein resorption; a3 disruption impacts ameloblasts/enamel (mouse)	Apical membrane of ruffle-ended ameloblasts; endosomal/lysosomal membranes	ATP-driven (H^+^/ATP ≈ 2–4; electrogenic)	autosomal recessive distal renal tubular acidosis (dRTA) from ATP6V1B1/ATP6V0A4 mutations; impaired acidification can disrupt matrix processing	[4,36,218,219,220]
H^+^/Na^+^	*SLC9A1* (NHE1)	Na^+^/H^+^ exchange (H^+^ efflux, Na^+^ influx)	Basolateral pH_i_ recovery/stabilization after acid loads; supports vectorial HCO_3_^−^ secretion (via CA-generated HCO_3_^−^ + H^+^ extrusion)	Basolateral	~1 Na^+^:1 H^+^ (electroneutral)	Not directly linked to AI	[1,177,212,221,222]
Na^+^/K^+^	*ATP1A1/ATP1B1* (Na^+^/K^+^-ATPase α1/β1)	Na^+^ out, K^+^ in	Maintains the Na^+^ gradient that powers secondary transport (NBCe1, NCKX/NCX, NHE1; supports CFTR/SLC26 circuits and cell-volume control)	Cytoplasmic and Basolateral membrane of secretory and maturation ameloblasts (also papillary layer)	3 Na^+^ out:2 K^+^ in (ATP)	Essential for epithelial transport; no enamel-specific AI link	[1,170,174]
Na^+^/K^+^/Cl^−^	*SLC12A2* (NKCC1)	Na^+^, K^+^, 2 Cl^−^ in	Osmolyte control and Cl^−^ supply from support layers to sustain ameloblast ion transport; regulatory volume function	Papillary layer and outer enamel epithelium (non-ameloblast support cells; SI/SR in earlier stage context)	1 Na^+^ + 1 K^+^ + 2 Cl^−^ in	Nkcc1^−^/^−^: late-maturation ameloblasts disorganized/shorter and ~10% lower enamel mineral density; ↑ Connexin 43 (Cx43)/NBCe1/SLC26A3/A6 compensation (hypomineralization)	[1,68,175]
K^+^	*KCNJ15* (Kir4.2)	K^+^ recycling	K^+^ uptake from enamel fluid; helps stabilize apical membrane potential during ruffle-ended phases; coordinates with NCKX4/Na^+^ handling	Apical border of ruffle-ended ameloblasts and cytosol of SA (reduced in smooth-ended; mislocalized with fluorosis/Wdr72 loss)	Channel (rectifier; non-stoichiometric)	No direct AI link; apical localization is reduced in fluorosis	[168,170]
Mg^2+^	CNNM4	Mg^2+^ efflux (Na^+^-linked)	Prevents intracellular Mg^2+^ buildup; supports proper crystal chemistry and maturation	Basolateral membrane of maturation ameloblasts	Na^+^-linked; stoichiometry not established in ameloblasts (2 Na^+^:1 Mg^2+^ reported in other cells)	Biallelic CNNM4 Mutations → Jalili syndrome (cone-rod dystrophy + AI)	[1,184,223,224]
Mg^2+^	TRPM7	Divalent-permeable channel (Mg^2+^ influx; also Ca^2+^)	Maintains Mg^2+^ homeostasis; supplies Mg^2+^ needed for TNAP/ALPL activity; also contributes to/positively modulates Ca^2+^ entry	Plasma membrane of maturation ameloblasts (evident in HAT-7 and mouse incisor; basolateral enrichment proposed)	Channel (non-stoichiometric)	TRPM7 kinase-dead or enamel-epithelium cKO → hypomineralized/hypoplastic enamel; reduced ALPase activity partly rescued by Mg^2+^	[185,225,226,227,228]
Citrate (Cit^3−^)	*SLC13A5* (NaCT)	Na^+^-coupled citrate influx	Supplies intracellular citrate for metabolism and incorporation at the mineral front; modulates crystal surface chemistry/toughness and transiently chelates Ca^2+^ during maturation	Basolateral (ameloblasts); papillary layer/support cells	Electrogenic ~3–4 Na^+^:1 citrate (pH-dependent)	Biallelic SLC13A5 variants → developmental and epileptic encephalopathy (DEE25); reported enamel hypoplasia/thin enamel	[229,230,231]
PO_4_^3−^	*ALPL* (TNAP)	Liberates PO_4_^3−^ by hydrolyzing PPi/other phosphomonoesters	Provides local orthophosphate for enamel mineral growth; supports matrix pH control	Stratum intermedium (high); also, maturation-stage ameloblasts	Enzyme (no fixed stoichiometry)	Hypophosphatasia (ALPL) → enamel defects/hypomineralization	[1,67,232,233]
PO_4_^3−^	*SLC53A1* (XPR1)	Pi efflux (non–Na^+^-coupled, IP_6_/InsP_7_-regulated)	Exports intracellular Pi toward the enamel space; coordinates with TNAP (PPi→Pi) and NaPi-IIb uptake to maintain matrix Pi/PPi balance	Apical (Tomes’ process, putative)	Not fully established; electrogenic (non-stoichiometric)	Biallelic XPR1 variants → primary familial brain calcification (PFBC/IBGC6); no enamel-specific phenotype	[11,233,234,235]
PO_4_^3−^	*SLC20A1/2* (PiT1/2)	Na^+^-coupled Pi influx	Pi import into ameloblasts (PiT1); PiT2 prominent in support layers → paracrine Pi supply	PiT1: ameloblasts (likely basolateral); PiT2: stratum intermedium/sub-odontoblastic layer	2 Na^+^:1 Pi (in)	No firm AI link; Slc20a2^−^/^−^ → dentin defects, enamel largely preserved	[1,9,236,237,238]
HPO_4_^2−^	*SLC34A2* (NaPi-IIb)	Na^+^-coupled Pi influx	Pi import to support mineral growth	Apical and nuclear in secretory; apical, and cytoplasmic in maturation	3 Na^+^:1 HPO_4_^2−^ (in)	Variants → pulmonary alveolar microlithiasis (PAM); no direct AI link	[9,10,29,239,240]
Fe	Ferritin (FTH1)	Iron storage (Fe^3+^ mineral core)	Sequesters Fe to limit oxidative stress; transient reservoir for Fe later deposited into enamel surface (rodent incisors)	Cytosol and ferritin-containing vesicles of late-maturation ameloblasts; papillary layer	Nanocage; up to ~4500 Fe atoms	Disrupted iron handling (e.g., ATG7 loss) → removes the iron deposit that normally pigments rodent incisors and can impair enamel, not classified as AI (hereditary)	[241,242,243,244,245]
F^−^	(Fluoride incorporation)	F^−^ substitution for OH^−^ in apatite	Forms fluorapatite; lowers crystal solubility and increases acid resistance	Enamel mineral lattice (surface-enriched)	1 F^−^:1 OH^−^ (Ca_10_(PO_4_)_6_(OH)_2_ → Ca_10_(PO_4_)_6_F_2_)	Excess during development → fluorosis; low exposure → ↑ caries risk	[154,246,247,248,249]
